# Genome-Wide Identification and Expression Profile Analysis of Citrus Sucrose Synthase Genes: Investigation of Possible Roles in the Regulation of Sugar Accumulation

**DOI:** 10.1371/journal.pone.0113623

**Published:** 2014-11-24

**Authors:** Mohammad Zahidul Islam, Xiao-Mei Hu, Long-Fei Jin, Yong-Zhong Liu, Shu-Ang Peng

**Affiliations:** Key Laboratory of Horticultural Plant Biology (Huazhong Agricultural University), Ministry of Education, Wuhan, People's Republic of China; Chinese Academy of Sciences, China

## Abstract

Sucrose synthase (Sus) (EC 2.4.1.13) is a key enzyme for the sugar accumulation that is critical to form fruit quality. In this study, extensive data-mining and PCR amplification confirmed that there are at least six Sus genes (*CitSus*1-6) in the citrus genome. Gene structure and phylogeny analysis showed an evolutionary consistency with other plant species. The six Sus genes contain 12–15 exons and 11–14 introns and were evenly distributed into the three plant Sus groups (*CitSus*1 and *CitSus*2 in the Sus I group, *CitSus*3 and *CitSus*6 in the Sus II group, and *CitSus*4 and *CitSus*5 in the Sus III group). Transcripts of these six CitSus genes were subsequently examined. For tissues and organs, *CitSus*1 and 2 were predominantly expressed in fruit juice sacs (JS) whereas *CitSus*3 and 4 were predominantly expressed in early leaves (immature leaves), and *CitSus*5 and 6 were predominantly expressed in fruit JS and in mature leaves. During fruit development, *CitSus*5 transcript increased significantly and *CitSus*6 transcript decreased significantly in fruit JS. In the fruit segment membrane (SM), the transcript levels of *CitSus2* and 5 were markedly higher and the abundant levels of *CitSus*3 and 6 gradually decreased. Moreover, transcript levels of *CitSus*1-4 examined were higher and the *CitSus*5 transcript level was lower in the fruit SM than in fruit JS, while *CitSus*6 had a similar transcript level in fruit JS and SM. In addition, transcripts of *CitSus*1-6 responded differently to dehydration in mature leaves or to mild drought stress in fruit JS and SM. Finally, the possible roles of Sus genes in the regulation of sugar accumulation are discussed; however, further study is required.

## Introduction

Sucrose is the principal form of photoassimilate for export from the source to sink organ in some plants, such as citrus [1]. The utilization of sucrose in the plant cell requires its cleavage, which is performed by two key enzymes, sucrose synthase (Sus, EC 2.4.1.13) and invertase (Inv). Of them, Sus catalyzes the reversible reaction of sucrose and UDP into UDP-glucose and fructose, whereas Inv hydrolyzes sucrose into glucose and fructose [2]. It is known that Sus plays pivotal roles in a variety of plant metabolic processes, such as sucrose distribution in plant tissues [3], [4], [5], [6], starch biosynthesis, cellulose synthesis and secondary cell-wall formation [7], response to abiotic stresses [3], [5], [8] and nitrogen fixation [9]. In citrus, Sus has shown its potential roles in fruit development and in promoting sugar accumulation in the juice sacs (JS) of grapefruit (*Citrus paradisi*) [10]. Moreover, Hockema and Etxeberria [5] suggested that the increase of sugar content in citrus fruit under drought stress is due to the increase in Sus activity that promotes photoassimilate partitioning into fruit JS. Our previous research on ‘Egan 1’ Ponkan (*C. reticulata* cv. Egan 1) showed that the significant increase of sugar accumulation in the juice sacs under soil plastic film mulch was attributed to the enhancement of Sus activity (cleavage direction) in the fruit segment membrane (SM) and Sus synthetic activity in fruit JS [3].

The identification of the genes encoding Sus is the first step towards understanding their physiological roles and involvement in different metabolic processes. To date, an increasing number of Sus gene families have been identified with the sequencing of the genomes of many plants. It is known that Sus isoforms are encoded by a small multi-gene family. For instance, the Sus family is comprised of six distinct members in the model plant *Arabidopsis thaliana* [11], *Oryza sativa* [12], *Gossypium arboreum* [13] and *Hevea brasiliensis* [8]. In addition, four Sus genes were found in *Hordeum vulgare* [14], whereas seven Sus genes were found in the genus *Populus* [15], [16]. In all cases, divergent expression patterns were examined in different isozymes of the respective Sus gene families, which implied that each member of Sus gene families has a particular function in a given tissue or organ of the species.

Citrus is an economically important crop globally that had an annual production exceeding 123.49 million tons in 2011 (FAOSTAT 2013). The regulation of sugar accumulation in fruits is very important for fruit quality improvement and drought tolerance [17], [18]. Although Sus has shown its potential roles in sugar accumulation and drought tolerance through the analysis of Sus enzyme activities [3], [5], [10] and each member of Sus gene families may have a specific function in a given tissue or organ of the species [11], [13], [14], [15], [16], [19], the possible roles of citrus Sus genes are still unclear.

Although three Sus genes (*CuSuSy*1, *CuSuSy*2 and *CuSuSyA*) were isolated from *C. unshiu* fruits by PCR, using a shuttle method a decade before [20], it was predicted that there would be more Sus genes in the citrus genome due to the number of Sus genes reported in other plants [8], [11], [12], [13]. Because citrus genome sequences have been published (http://citrus.hzau.edu.cn/orange/ and http://www.phytozome.net/), it is possible to identify more citrus Sus genes. In the present study, we succeeded in identifying six citrus Sus genes based on the citrus genome sequence and investigated their temporal-spatial expression patterns in different tissues or organs, in fruit JS (sugar-stored tissue) and in SM (sucrose-downloaded tissue) during fruit development and ripening and under drought treatment. These comprehensive results are fundamentally important for the understanding of the possible roles of Sus genes in sucrose transport or sugar accumulation in citrus fruit.

## Materials and Methods

### Plant materials

Healthy and uniform fruits were sampled at during a rapid growth period [106 days after anthesis (DAA)] and a ripening period (165 DAA) from 15-year-old ‘Guoqing No. 1’ Satsuma mandarin (*Citrus unshiu* cv. Guoqing No. 1) grafted on *Poncirus trifoliata* in the citrus orchard of Huazhong Agricultural University. SM and JS from each fruit were separated on the ice-containing pot. The samples were frozen using liquid nitrogen immediately after collection and then stored at −80°C until further use.

Fruit JS at 120 DAA, flower (FL, just in a white bud stage, not blooming), early leaf (EL, the length is 2 cm or so) and mature leaf (ML, the length is 10 cm or so) samples were collected independently from a ‘Guoqing No. 1’ Satsuma mandarin tree. Samples were frozen using liquid nitrogen and stored at −80°C for gene-tissue expression analysis.

### Dehydration or drought treatment

To perform the water stress treatment, 30 healthy, uniform MLs (average length 11 cm) were collected and dehydrated on filter paper at 20°C for 0 to 10 h. At 0 h, 2 h and 10 h after dehydration (HAD), 10 leaves were randomly selected, frozen in liquid nitrogen, and stored at -80°C for RNA extraction. Healthy and uniform fruits of control and drought-treated trees were collected from ‘Guoqing No. 1’ trees at 60 days after film mulch. SM and JS were separated, frozen in liquid nitrogen immediately, and then stored at −80°C until further use. Mild drought stress (MDS, no obvious phenotypic change was observed in the leaf and other tissues) was created using a film mulch on the soil, performed during the rainy season, as previously described [3].

### Mining of citrus sucrose synthase genes

To predict the Sus homologs in citrus, the sequence of *CuSuSy1* or *CuSuSyA* as reported by Komatsu et al. [20] were used as a query to search in three citrus genome databases [one sweet orange genome database [21] from Huazhong Agricultural University (HZAU), China (citrus.hzau.edu.cn/orange), and the others, including sweet orange and Clementine genome databases, from Phytozome (www.phytozome.net)]. The filter criteria were that the E-value is zero or near zero and the sequence annotation is the target gene's name. DNASTAR Lasergene Software (USA) was employed to compare their identities, pIs and molecular weights. After grouping, gene-specific primers (Table S1) were designed using the program Primer 3.0 [22] based on their respective genomic sequences for PCR to identify their authenticity. The PCR amplification conditions were 94°C for 2 min, followed by 30 cycles of 94°C for 45 s, 60°C for 1 min, 72°C for 1 min, and then a final 10-min extension at 72°C. Amplification products were cloned into the pMD18-T cloning vector (TaKaRa Biotechnology, Dalian, China) and then transformed into *E.coli* competent cells (DH5α) for sequencing. Gene structure analysis of citrus Sus genes was performed by using the Gene Structure Display Server (GSDS, gsds.cbi.pku.edu.cn)[23]. Conserved domains (CD) were searched in the conserved domain database by using the batch CD-searching program [24].

### Phylogenetic analysis

Phylogenetic or molecular evolutionary analysis was constructed by MEGA 4.0 using neighbor-joining methods [25]. Bootstrap analysis was performed using 1000 replicates. Gene or protein accession numbers containing citrus Sus genes and other known Sus genes used in this study are listed in Table S2.

### Quantitative real-time PCR analysis

Total RNA of all samples was isolated according to a previously described protocol [26]. Five µg of high-quality total RNA was treated using DNase I (Fermentas) at 37°C for 1 h, and then was used for the first-strand cDNA synthesis using the RevertAid M-MuLV Kit (Fermentas). Specific primers designed by Primer 3.0 [22] for quantitative Real-Time PCR (qRT-PCR) are listed in Table 1. Actin was used as an internal control to normalize the expression level of the target gene among different samples. Additionally, prior to qRT-PCR, the amplifying products of ‘Guoqing No. 1’ mandarin with each pair of primers were sequenced and it was confirmed that they belonged to their respective Sus genes. The qRT-PCR was conducted in three biological replicates. qRT-PCR was performed in a 10 µL reaction volume using the Thunderbird SYBR qPCR Mix (TOYOBO, JAPAN) on the LightCycler 480 Real Time System (Roche, Switzerland) following the manufacturer's protocol. Reactions started with an initial incubation at 50°C for 2 min and at 95°C for 4 min, then 45 cycles of 95°C for 15 s, 58°C for 10 s and 72°C for 20 s. The Livak method [27] was employed to calculate the relative gene expression level.

**Table 1:** Specific primers for quantitative real-time PCR.

Putative gene name	Sequence (5′-3′)		Amplicon size (bp)
	Forward primer	Reverse primer	
***CitSus1***	CTGGAGGTGGGGGTAGGTTTA	ATCCTTGACAAAAGGGCCAAGA	291
***CitSus2***	GAACTTACAAGCGGCAGCAG	CACCGAGATCTCCTCAACATCA	197
***CitSus3***	CACCGCTCCATCCTAACTCG	ATCCCTTTGCCTTGAGCCAC	299
***CitSus4***	ACAGCTAGCGTTCTCAGTTCA	AAGCCCTCTAACACCTTGCC	291
***CitSus5***	ACGAAGCTTAATCAATTCTTGCT	AGCTTCCTGCGTAGAACACA	295
***CitSus6***	ACACTCTCGCTTCTCACTACG	CATGAAAGGGCTCTTGCTGA	270
***actin***	CCGACCGTATGAGCAAGGAAA	TTCCTGTGGACAATGGATGGA	190

### Statistical analysis

Differences between samples, if needed, were evaluated by a t-test or Duncan's test at P = 0.05.

## Results

### Data mining, isolation and molecular characterization of the citrus sucrose synthase gene family

To detect potential Sus homologs in citrus, an extensive database search was performed in the three citrus genome databases using the sequence of *CuSuSy1* or *CuSuSyA* as reported by Komatsu et al. [20]. Queries with either *CuSuSy*1 or *CuSuSyA* produced the same results indicating that there were at least 6 Sus genes in either the sweet orange or the Clementine genome database. Their transcript IDs are listed in Table 2. Based on their putative size, pI and identity, these transcripts can be divided into six groups. In each group, their mutual sequence identities were almost all more than 99%. The sequence of Ciclev10024638 m is truncated, so the identity with the other two sequences was relatively low (approximately 85.0%).

**Table 2:** Transcripts encoding for sucrose synthase in three citrus genome databases and their pairwise identities.

Putative Name	Transcript ID#	Protein size (Amino acid)	Mol. wt (KDa)	pI	Identity (%)	
					Or-	Ci-
***CitSus1***	Cs4g06850.1	805	92.2	6.11	100	99.9
	orange1.1g003661m	805	92.2	6.11		99.9
	Ciclev10007483m	806	92.3	6.06		
***CitSus2***	Cs4g06900.1	780	89.2	5.97	97.3	98.8
	orange1.1g003947m	784	89.4	5.90		98.2
	Ciclev10010343m	780	89.1	5.97		
***CitSus3***	Cs5g33470.1	811	92.6	6.23	99.9	99.6
	orange1.1g003492m	816	93.2	6.29		99.8
	Ciclev10018889m	811	92.6	6.23		
***CitSus4***	Cs5g16700.1	867	98.2	7.83	99.3	85.0
	orange1.1g002909m	867	98.1	7.84		85.6
	Ciclev10024638m	326	37.2	9.26		
***CitSus5***	Cs6g15930.1	839	95.3	6.13	99.7	99.3
	orange1.1g036539m	749	84.9	5.37		99.3
	Ciclev10011062m	839	95.2	6.17		
***CitSus*** **6**	Cs9g03980.1	808	92.6	6.20	99.8	100
	orange1.1g003726m	800	91.7	6.13		99.8
	Ciclev10004341m	808	92.6	6.20		

# Transcript ID with ‘Cs’ as the beginning two letters derives from the sweet orange genome database at Huazhong Agricultural University (China, http://citrus.hzau.edu.cn/orange/). Transcript ID with ‘or’ as the beginning two letters derives from the sweet orange genome database in Phytozome (http://www.phytozome.net/). Transcript ID with ‘Ci’ as the beginning two letters derives from the Clementine genome database in Phytozome. Identity was produced by alignment with the Clustal W method.

The six putative Sus genes were named *CitSus*1 to 6 (Table 2). Based on the transcript sequences from the sweet orange genome database of HZAU, we designed six pairs of primers and succeeded in amplifying specific bands by using the first-strand cDNA of *C. unshiu* fruit as a template. Sequencing results showed that the identity of each PCR product was more than 98% with its respective genome transcript sequence (Table S1), which confirmed the authenticity of the Sus transcripts in the citrus genome. Therefore, transcript sequences from the HZAU citrus genome database were used in the following sequence analysis.

**Figure 1:**
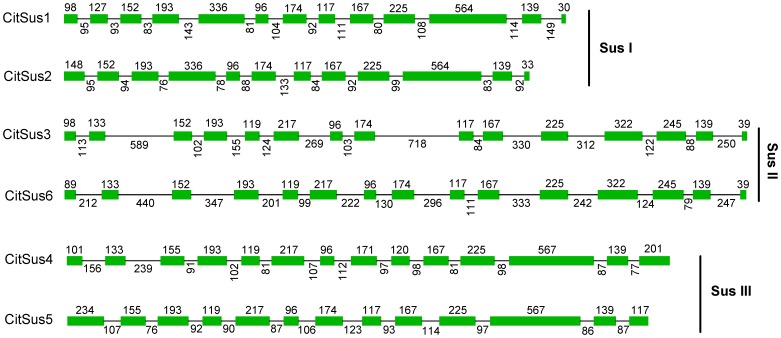
Schematic representation of the six Sus gene structures in citrus. Boxes indicate exons and single lines between the boxes indicate introns. The numbers on boxes and down lines indicate the length of the exon and intron, respectively. Sus I, II and III refer to the three groups of Sus genes family shown in Figure 2.

The identities between citrus Sus genes were from 54.4% (between *CitSus*2 and *CitSus*4) to 80.7% (between *CitSus*3 and *CitSus*6) at their amino acid sequence levels. Moreover, CitSus1 had a 98.9% and 98.6% amino acid identity with CuSuSy1 and CuSuSy2, respectively, while CitSus3 had a 99.9% amino acid identity with CuSuSyA (Table S3). The peptide sequences of these six putative Sus genes contain 780–867 amino acids, 5.97–7.83 predicted isoelectric points, and molecular weights from 89.2 to 98.2 kDa (Table 2). Similar to other plant Sus gene families, batch CD-search indicated that the six citrus Sus genes contained three domain families (GT1_Sucrose_synthase, Glycosyltransferase_GTB_type superfamily and multi domains of PLN00142) (Table S4). Moreover, full-length cDNA and gDNA sequences of all six citrus Sus genes were downloaded from the *C. sinensis* genome database (citrus.hzau.edu.cn/orange/). Gene structure analysis showed that the six Sus genes contain 12–15 exons and 11–14 introns. Specifically, *CitSus*2 contains 12 exons and 11 introns; *CitSus*1 and *CitSus*5 contain 13 exons and 12 introns; *CitSus*3 and *CitSus*6 contain 15 exons and 14 introns; and *CitSus*4 contains 14 exons and 13 introns. The sizes of five exons were conserved while the sizes of the introns were absolutely different among the six citrus Sus genes (Figure 1).

**Figure 2:**
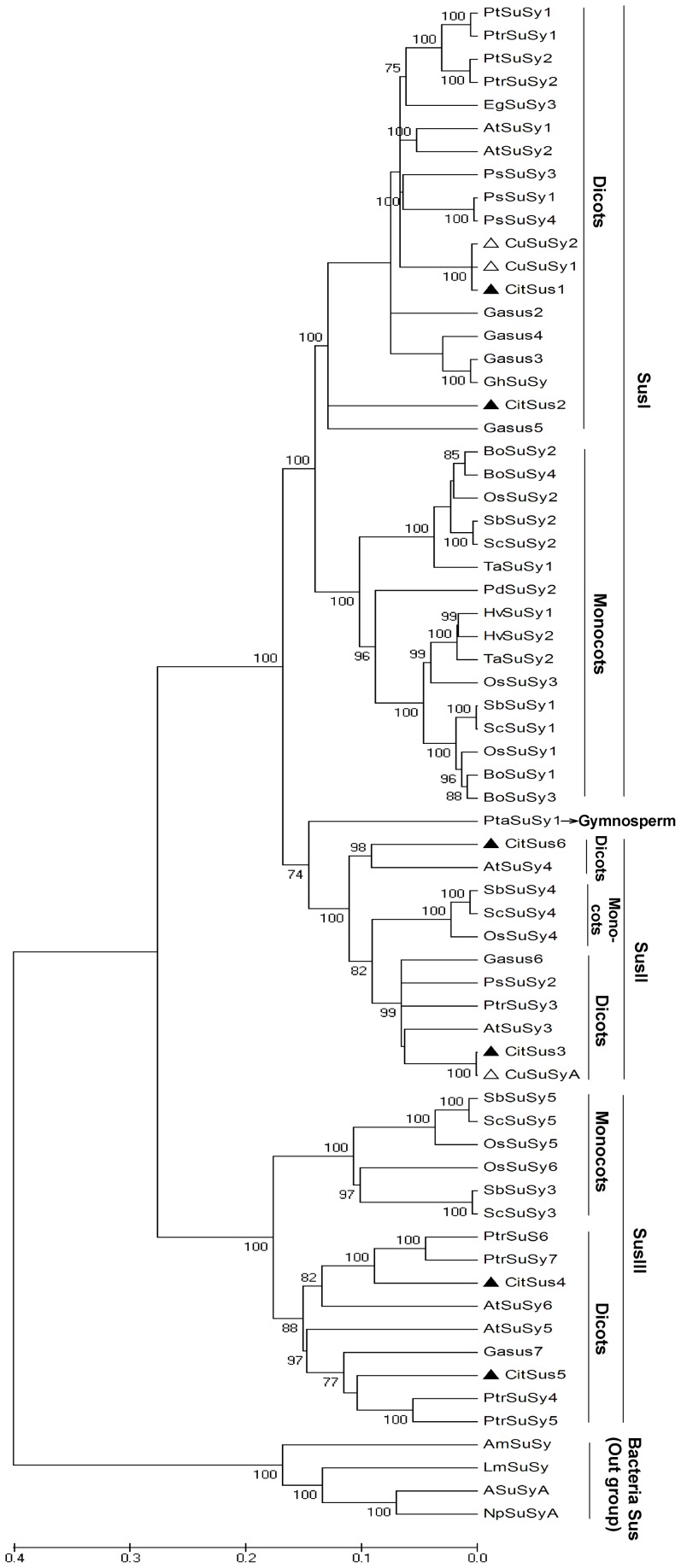
Phylogenetic analysis of citrus and other plant Sus homologs. The phylogenetic tree was constructed by the MEGA 4.0 program with the neighbor-joining method. Sus accession numbers are listed in Table S2. The black triangle shows the position of six citrus Sus isoforms. The white triangle shows the position of three *c. unshiu* Sus isoforms reported by Komatsu *et al.* [20].

In order to investigate the relationship between *CitSus* genes and other plant Sus homologs, 36 sequences from dicot species, one sequence from gymnosperm, 25 sequences from monocot species, and four sequences from bacteria species were used to construct a phylogenetic tree with MEGA 4.0 software using the neighbor-joining method (Figure 2). As shown in Figure 2, all the Sus genes were clustered into four groups. Of these, 61 plant Sus genes were clustered into three major groups, named Sus I, II and III, while four bacterial Sus genes were clustered into the same group (out group). In addition, the Sus I and III groups could be further classified into two distinct sub-groups, consisting exclusively of dicot Sus proteins and monocot Sus proteins, respectively. Sus II may be further subcategorized into two dicot Sus proteins and one monocot Sus. One gymnosperm Sus could be clustered into the Sus II group. The six citrus Sus isozymes were evenly distributed into the three plant Sus groups: *CitSus*1 and *CitSus*2 in the dicot sub-group of Sus I, *CitSus*3 and *CitSus*6 in the dicot sub-group of Sus II, and *CitSus*4 and *CitSus*5 in the dicot sub-group of Sus III. In addition, *CitSus1* was clustered together with *CuSuSy1* and *CuSuSy2* whereas *CitSus3* was closer to *CuSuSyA*. Moreover, the *CitSus*2 was closer to *Gossypium arboretum* Sus5; *CitSus*4 was close to *Populus trichocarapa* Sus 6 and 7 while *CitSus*5 was relatively close to *G. arboretum* Sus7 and *P. trichocarapa* Sus 4 and 5; and the *CitSus*6 was closer to *A. thaliana SuSy*4 (Figure 2).

### Expression analysis of Citrus Sus genes in different tissues

**Figure 3:**
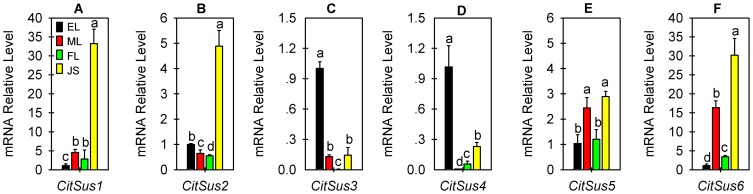
Relative transcript levels of six citrus Sus genes in different citrus tissues or organs. The expression levels of the six citrus Sus genes were measured by real-time qRT-PCR and standardized by actin gene expression level. The total RNA was extracted from early leaf (EL), mature leaf (ML), flower (FL) and fruit juice sacs (JS) (details in Materials and Methods). All qRT-PCR values are the average ±Se of three replicates. Bars marked with lower-case letters indicate that the expression levels showed significant difference at P<0.05 by using Duncan's test.

**Figure 4:**
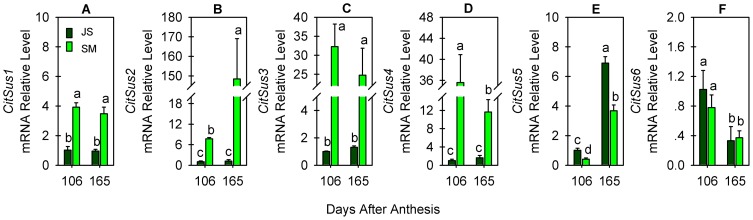
Relative transcript levels of *CitSus*1-6 in fruit juice sacs (JS) and segment membrane(SM) at 106 and 165 days after anthesis. A-F refer to the transcript levels of *CitSus*1-6 genes, respectively. qRT-PCR values are the average ±Se of three replicates. Bars marked with lower-case letters indicate that the gene expression levels showed significant difference at P<0.05 by using Duncan's test.

Expression patterns of six citrus Sus genes were first examined in different tissues, including EL, ML, FL and JS (Figure 3 and 4). *CitSus1* was expressed predominantly in JS, which was more than 30-, 6- and 10- times higher than that in EL, ML and FL, respectively (Figure 3A). Similar with *CitSus1*, *CitSus2* was also expressed predominantly in JS, but was only approximately 5-, 7- and 10-times higher than that in EL, ML and FL, respectively (Figure 3B). Different from *CitSus1* and *CitSus2*, both *CitSus3* (Figure 3C) and *CitSus4* (Figure 3D) were highly expressed in EL, which were more than 3 times higher than that of other three tissues; however, *CitSus3* and *CitSus4* transcript levels were very low in FL and ML, respectively (Figure 3C and D). As for *CitSus5*, its transcript levels were similar in EL and FL, or in ML and JS, however, the transcript levels in ML and JS were more than 2-fold higher than those in EL and FL (Figure 3E). Similar with *CitSus1*, *CitSus6* transcript level was detected predominantly in JS, which was more than 30-, 2- and 12-times higher than that in EL, ML and FL, respectively (Figure 3F).

The detection of enzyme activity showed that Sus plays an important role in sugar accumulation in citrus fruit [3], [5], [10]. Here, the transcript levels of six citrus Sus genes were examined in two development stages (fruit rapid growth and ripening) of edible tissues [JS and SM] (Figure 4). Transcript levels of *CitSus*1-4 in JS were obviously lower than those in SM (Figure 4 A-D) while the *CitSus*5 transcript level was significantly higher in JS than in SM (Figure 4E) and the *CitSus*6 transcript level in JS was similar with that in SM (Figure 4F) at any development stage. Specifically, *CitSus1* transcript did not change obviously between the two developmental stages either in JS or in SM but its transcript level in JS was less than one third of that in SM (Figure 4A). Different from *CitSus1*, the *CitSus*2 transcript level did not change obviously in JS, but it was increased significantly in SM as the fruit ripened, levels were more than 15 times higher at 165 DAA than that at 106 DAA (Figure 4B). *CitSus*3 and *CitSus*4 transcripts showed a similar changing trend from 106 DAA to 165 DAA either in JS or in SM; a slight increasing trend in JS and a decreasing trend in SM (Figure 4C and D) was observed; the *CitSus*3 transcript level decreased slightly in SM but the *CitSus*4 transcript level was decreased significantly in SM and, at 165 DAA, it was approximately one-third of that at 106 DAA (Figure 4D). Different from the changes of the *CitSus*3 and *CitSus*4 transcripts, *CitSus*5 and *CitSus*6 transcripts showed a reverse changing trend from 106 DAA to 165 DAA either in JS or in SM: *CitSus*5 transcript level was increased significantly while *CitSus*6 transcript was decreased significantly from 106 DAA to 165 DAA either in JS or in SM (Figure 4E and F). At 165 DAA, the *CitSus*5 transcript level (Figure 4E) was more than 7 times higher, while the *CitSus*6 transcript level (Figure 4F) was approximately half of that at 106 DAA either in JS or in SM.

### Expression analysis of Citrus Sus genes in response to dehydration or mild drought stress

**Figure 5:**
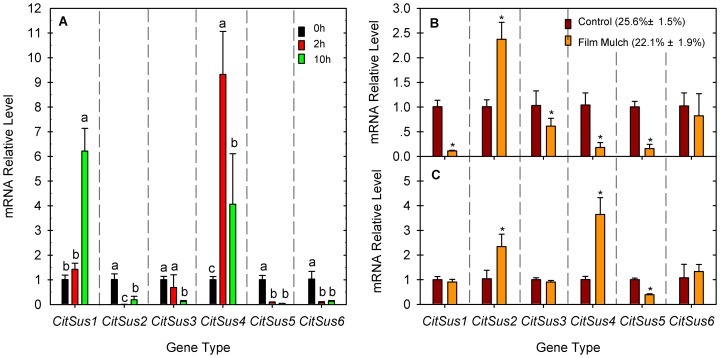
Relative transcript levels of *CitSus*1-6 in mature leaves in response to dehydration (A) and in fruit segment membrane (B) and juice sacs (C) in response to mild drought stress. Bars marked with lower-case letters indicate that the expression levels showed significant difference at P<0.05 by using Duncan's test. Asterisk (*) on bars indicates the gene expression levels showed significant difference between film mulch and the control at *P*<0.05 by using *t*-test (LSD). In the present study, film mulch decreased the soil water content creating a mild drought stress compared with the control.

The transcript levels of *CitSus*1-6 genes were examined in ML in response to dehydration as well as in fruit SM and JS under MDS (Figure 5). In the ML (Figure 5A), *CitSus*1 and *CitSus*4 transcripts were significantly increased while transcripts of the other citrus Sus genes were obviously reduced by dehydration. Under dehydration, the *CitSus*1 transcript level progressively increased and it was more than 6-fold higher at 10 HAD than at 0 HAD; *CitSus*4 transcript level was rapidly increased to 9 times at 2 HAD and decreased 4 times at 10 HAD, compared with that at 0 HAD. In contrast, transcript levels of *CitSus* 2, 3, 5 and 6 at 2 and 10 HAD were one-fifth less of that at 0 HAD, respectively, except for the transcript level of *CitSus*3 at 2 HAD, which was just slightly reduced compared with that at 0 HAD (Figure 5A).

In fruit SM (Figure 5B), transcript levels of *CitSus*1 and *CitSus* 3–5 were significantly reduced and the *CitSus*6 transcript level was slightly reduced while only the *CitSus*2 transcript level was increased nearly 2.5 times the transcript level with MDS compared with that of the control. In fruit JS (Figure 5C), *CitSus*2, *CitSus*4 and *CitSus*6 transcripts showed an increasing trend in which the *CitSus*2 transcript level was increased up to 2 times and *CitSus*4 transcript level was increased up to 3.6 times compared with the control, significantly higher than their control. In contrast, *CitSus*1, *CitSus*3 and *CitSus*5 transcript levels showed a decreasing trend of which only *CitSus*5 transcript level was significantly decreased in the MDS treatment.

## Discussion

It is clear that Sus isoforms are encoded by a small multi-gene family [11]. Similar with *A. thaliana* [11], *O. sativa* [12], *G. arboreum* [13] and *H. brasiliensis* [8], we discovered that there are also at least six Sus gene members in the citrus genome (Table 2) by searching sweet orange and Clementine genomic databases and with PCR amplification. Similarity analysis of the partially cloned *CitSus*1-6 from *C. unshiu* fruits showed the highest identity (over 98%) with the genomic sequence (Table S1) and batch CD-search indicated that they also have two typically conserved domains of Sus polypeptide (GT1_Sucrose_Synthase and Glycosyltransferase_GTB_type superfamily) (Table S4), which confirmed their authenticity in the citrus genome.

A comprehensive analysis of gene structure, including exon/intron number and position, leads to some conclusions regarding the possible origin, relationships and predicted functions among the different Sus isomers. Our present study supported the idea that the characteristic features of these six putative *CitSus* protein sequences and their exon/intron structure (Figure 1) were highly conserved to the Sus orthologs of other plant species [15]. In the structural evolutionary history of gene families, exon/intron insertion or deletion or both events between the paralogs may be happening to some extent and represent attribution features [28]. In the present study, the number and position of introns of six putative Sus genes were different in size but showed parallel positions and were flanked by GT-AG boundaries, highly consistent with rubber [8], poplar [15] and cotton [13]. In addition, we also found that the six putative *CitSus* isozymes were evenly distributed into the three plant Sus groups (SusI, SusII and SusIII) (Figure 2), similar with other plant Sus gene distribution [8], [11], [13], [15], [19]. Moreover, the three *C. unshiu* Sus genes identified by Komatsu et al. [20] were clustered into two Sus dicot sub-groups in the present study, similar to a previously constructed phylogenetic tree [20].

It has been reported that Sus plays an important role in different metabolic processes [3], [4], [7], [9]. Recently, remarkable divergence was found in gene structure and gene expression patterns among the Sus gene family in many plants, such as Arabidopsis [11], [29], barley [14], rice [12], cotton [13] and poplar [15]. This divergence was considered to be related to their functional diversity [7], [30]. In this study, we also demonstrated that the six citrus putative Sus genes have different spatio-temporal expression patterns (Figure 3 and 4), which may reflect the functional difference of Sus genes in citrus.

Sucrose, the main-exporting photoassimilate, is synthesized in the ML and is then transported to the EL, FL, fruit and other organs or tissues. In general, ML belongs to the source organ responsive for sucrose synthesis and acts as a sucrose-exporting center whereas EL, FL and fruit JS belong to sink organs, which receive sucrose for their development or storage [31]. Citrus Sus activity includes two directions: a synthetic direction and a cleavage direction. Schaffer et al. [32] measured Sus activity during leaf development of *C. sinensis* cv. Shamouti and found that Sus activity of the synthetic direction was increased significantly while Sus activity of the cleavage direction was reduced, suggesting the major role of Sus is to provide UDP-glucose for synthesis of cell wall polysaccharides in the expanding leaf and to synthesize sucrose for export in the mature leaf. Here, we found that the transcript levels of *CitSus*2-4 in ML were significantly decreased from those of EL (Figure 3B–D). Moreover, the transcript level of *CitSus*3 was more than 10-times higher in EL than in ML (Figure 3C) and *CitSus*4 transcript was almost undetectable in ML (Figure 3D). In addition, transcript levels of *CitSus*1, 5 and 6 in ML were significantly increased to more than 5, 2.5 and 15 times, respectively, compared with that in the EL (Figure 3A, E and F). These results suggested that *CitSus*2-4 may play major roles in immature leaves while *CitSus*1, 5 and 6 may play major roles in ML.

Fruit flesh is physically separated into two tissues: transport tissue (vascular bundles and SM) and phloem-free JS. SM owns three vascular bundles and is the only site for the entry of assimilate into JS [10]. It is well known that sucrose partitioning into fruit is mainly determined by its sink strength, which is the competitive ability of an organ to attract assimilates and is preferentially related to the ability of sucrose-metabolizing enzymes to hydrolyze sucrose, such as Sus [2], [10]. A previous study was carried out to detect Sus activities in Ponkan fruits and, the results showed that changes in Sus activity varied between fruit SM and JS during fruit development and ripening [3]: in fruit SM, Sus activity of the cleavage direction stayed at a relatively high level and showed only a slight change while Sus activity of the synthetic direction was slightly lower but increased continuously; in JS, Sus activity of the cleavage direction was increased, although it was slightly lower than Sus activity of the synthetic direction, which kept a relatively constant level during fruit development and ripening. *CitSus* gene expression profiles in the present study revealed that *CitSus*1, 2, 5 and 6 were predominantly expressed in fruit JS (Figure 3A, B, E and F) and transcript levels of *CitSus*1-4 were significantly higher in fruit SM compare to JS (Figure 4A–D). In addition, it is possible that *CitSus*2 (Figure 4B) and/or *CitSus*5 and 6 (Figure 4E and F) play more roles in sucrose download and partitioning in fruits because their transcript levels were significantly changed in SM and/or JS from 106 DAA to 165 DAA.

It was clearly observed that drought stress can increase sugar accumulation in fruit [17], [18], [33]. Sugars help to maintain osmotic balance under dehydration or drought conditions [34]. Moreover, Hockema and Etxeberria [5] suggested that drought could enhance sink strength by increasing Sus activity and promoting photoassimilate partitioning into fruit JS. Film mulch on soil in the rainy season often creates MDS in fruit crops, which will increase sugar accumulation in fruit JS [3], [35], [36]. Jiang et al. [3] further reported that the increase of sugar accumulation in the fruit JS under MDS condition is due to the significant increase of the Sus activity (cleavage direction) in fruit SM along with the significant decrease of the Sus activity (both cleavage and synthetic directions) in fruit JS. The changes in Sus activities under MDS are mostly attributed to the effect of MDS on Sus gene transcript levels because the expression of some Sus genes or Sus proteins has been generally observed to be up-regulated in response to dehydration/drought stress [37], [38], [39]. Additionally, a recent report showed that drought treatment conspicuously induced *HbSus*5 expression in roots and leaves [8]. In our study, members of the citrus Sus family exhibited different responses of expression patterns in the ML in response to dehydration as well as in fruit SM and JS to MDS (Figure 5). The diverse responses of putative *CitSus* transcripts to drought suggested that their divergent roles in response to abiotic stresses are tissue-dependent. Moreover, although there are significant changes in some citrus Sus genes in fruit SM and JS, it is still difficult to establish relationships with the changes of Sus activity of either direction in fruit SM or JS under MDS condition.

## Conclusions

The current study is the first to identify the citrus Sus gene family through genome-scale searching, evolutionary and gene structure analysis, and the spatio-temporal expression patterns of each Sus member in woody-fruit crops. These results provide an underlying foundation and framework for future understanding of the potential physiological roles of each CitSus gene member involved in sugar accumulation during fruit development and in response to abiotic stresses, such as drought. The identification of entire Sus genes in citrus and their temporal-spatial expression profiles suggest that the function of each CitSus gene is tissue-dependent during fruit development or in response to abiotic stress. For better understanding of the specific function of each CitSus gene and their possible functional interactions, analysis with knockout mutants or gene suppression and gene over expression is required in future studies.

## Supporting Information

Table S1 Specific primers for the confirming PCR and sequence identities with respective transcript ID sequences.
(DOC)

Table S2 List of sucrose synthase gene sequences used in this study.
(DOC)

Table S3 Identity matrix for the amino acid sequences of six CitSus genes and three CuSuSy genes.
(DOC)

Table S4 Results of NCBI batch CD-search of six citrus sucrose synthases.
(DOC)
