# Supplementary material for: Genome-Wide Identification and Expression Profile Analysis of Citrus Sucrose Synthase Genes: Investigation of Possible Roles in the Regulation of Sugar Accumulation
Source: PLoS One. 2014 Nov 24;9(11):e113623. doi: 10.1371/journal.pone.0113623 (PMC4242728; doi:10.1371/journal.pone.0113623)
Supplement: Table S1 — Specific primers for the confirming PCR and sequence identities with respective transcript ID sequences. (DOC) [file pone.0113623.s001.doc]

Table S1 Specific primers for the confirming PCR and sequence identity with respective transcript ID sequence

| Transcript ID | Forward Primer | Reverse Primer | Identity* |
| --- | --- | --- | --- |
| Cs4g06850.1 | ATGGCAAGATTGGACCGTGT | ATCGCAATCCCAGAAGGGAC | 100% |
| Cs4g06900.1 | GTGGTACGGGAAGAACGCTA | AACATAGAGATGGCCCCACA | 98% |
| Cs5g33470.1 | ATAGGGCACGTAATGGCGAG | ACGGGAGGTAGCTGGGTAAT | 99% |
| Cs5g16700.1 | TCAGGCTTCCACATTGACCC | GGGTCCAAAGGTCACGTACA | 99% |
| Cs6g15930.1 | GTGACAGGCTCAGAAACGGA | TGGCTTGTGGTGAAATCCGA | 99% |
| Cs9g03980.1 | TTGCATGGCCAGTTTCGTTG | CTCGTCGACAGGGAGTTTCC | 99% |
| *The identity refers to the identity of PCR product sequence with the original nucleotide sequence in the genome database. First-strand cDNA of *Citrus unshiu* fruit was used as PCR template. | | | |
