# Supplementary material for: Genome-Wide Identification and Expression Profile Analysis of Citrus Sucrose Synthase Genes: Investigation of Possible Roles in the Regulation of Sugar Accumulation
Source: PLoS One. 2014 Nov 24;9(11):e113623. doi: 10.1371/journal.pone.0113623 (PMC4242728; doi:10.1371/journal.pone.0113623)
Supplement: Table S2 — List of sucrose synthase gene sequences used in this study. (DOC) [file pone.0113623.s002.doc]

| Table S2 List of sucrose synthase gene sequences used in this study | | | | |
| --- | --- | --- | --- | --- |
| Taxon | | Sequence name | Accession number | Protein size |
| Dicot plants | |  |  |  |
|  | *Citrus sinensis* | CitSus1 | Cs4g06850.1 | 803 |
|  |  | CitSus2 | Cs4g06900.1 | 780 |
|  |  | CitSus3 | Cs5g33470.1 | 811 |
|  |  | CitSus4 | Cs5g16700.1 | 839 |
|  |  | CitSus5 | Cs6g15930.1 | 839 |
|  |  | CitSus6 | Cs9g03980.1 | 808 |
|  | *Citrus unshiu* | CuSuSy1 | AB022092 | 805 |
|  |  | CuSuSy2 | AB029401 | 805 |
|  |  | CuSuSyA | AB022091 | 811 |
|  | *Arabidopsis thaliana* | AtSuSy1 | At5g20830 | 808 |
|  |  | AtSuSy2 | At5g49190 | 808 |
|  |  | AtSuSy3 | At4g02280 | 809 |
|  |  | AtSuSy4 | At3g43190 | 807 |
|  |  | AtSuSy5 | At5g37180 | 836 |
|  |  | AtSuSy6 | At1g73370 | 942 |
|  | *Gossypium arboreum* | Gasus2 | JQ995523 | 798 |
|  |  | Gasus3 | JQ995524 | 805 |
|  |  | Gasus4 | JQ995525 | 806 |
|  |  | Gasus5 | JQ995526 | 796 |
|  |  | Gasus6 | JQ995527 | 809 |
|  |  | Gasus7 | JQ995528 | 824 |
|  | *Pisum sativum* | PsSuSy1 | AJ012080 | 806 |
|  |  | PsSuSy2 | AJ001071 | 809 |
|  |  | PsSuSy3 | AJ311496 | 804 |
|  |  | PsSuSy4 | AF079851 | 806 |
|  | *Gossypium hirsutum* | GhSuSy | U73588 | 805 |
|  | *Populus tomentosa* | PtSuSy1 | GU559727 | 805 |
|  |  | PtSuSy2 | GU559728 | 803 |
|  | *Populus trichocarapa* | PtrSuSy1 | GU559729 | 805 |
|  |  | PtrSuSy2 | GU559730 | 803 |
|  |  | PtrSuSy3 | GU559731 | 811 |
|  |  | PtrSuSy4 | GU559732 | 815 |
|  |  | PtrSuSy5 | GU559733 | 835 |
|  |  | PtrSUuS6 | GU559734 | 800 |
|  |  | PtrSuSy7 | GU559735 | 810 |
|  | *Eucalyptus grandis* | EgSuSy3 | DQ227994 | 805 |
| Gymnosperms | |  |  |  |
|  | *Pinus taeda* | PtaSuSy1 | EF619967 | 833 |
| Monocot plants | |  |  |  |
|  | *Oryza sativa* | OsSuSy1 | Os03g0401300 | 816 |
|  |  | OsSuSy2 | Os06g0194900 | 808 |
|  |  | OsSuSy3 | Os07g0616800 | 816 |
|  |  | OsSuSy4 | Os03g0340500 | 809 |
|  |  | OsSuSy5 | Os04g0309600 | 844 |
|  |  | OsSuSy6 | Os02g0831500 | 846 |
|  | *Sorghum Bicolor* | SbSuSy1 | Sb01g033060 | 816 |
|  |  | SbSuSy2/SbSus2 | FJ513325 | 837 |
|  |  | SbSuSy3 | Sb04g038410 | 838 |
|  |  | SbSuSy4 | Sb01g035890 | 809 |
|  |  | SbSuSy5 | Sb10g031040 | 892 |
|  | *Saccharum** | ScSuSy5 |  | 866 |
|  |  | ScSuSy2 |  | 802 |
|  |  | ScSuSy3 |  | 824 |
|  |  | ScSuSy1 |  | 816 |
|  |  | ScSuSy4 |  | 806 |
|  | *Triticum aestivum* | TaSuSy1 | AJ001117 | 807 |
|  |  | TaSuSy2 | AJ000153 | 815 |
|  | *Hordeum vulgare* | HvSuSy1 | X69931 | 816 |
|  |  | HvSuSy2 | Y15802 | 823 |
|  | *Potamogeton distinctus* | PdSuSy2 | AB193516 | 842 |
|  | *Bambusa oldhamii* | BoSuSy1 | AF412036 | 816 |
|  |  | BoSuSy2 | AF412038 | 808 |
|  |  | BoSuSy3 | AF412037 | 816 |
|  |  | BoSuSy4 | AF412039 | 808 |
| C. Bacteria | |  |  |  |
|  | *Anabaena* | ASuSyA | AJ010639 | 806 |
|  | *Nostoc punctiforme* | NpSuSyA | AJ316589 | 806 |
|  | *Lyngbya majuscule* | LmSuSy | AY522504 | 804 |
|  | *Acaryochloris marina* | AmSuSy | NC_009925 | 807 |
| *Amino acid sequence of ScSusy 1to5 were from the study of Zhang et al.,2013 | | | | |
