# Supplementary material for: Genome-Wide Identification and Expression Profile Analysis of Citrus Sucrose Synthase Genes: Investigation of Possible Roles in the Regulation of Sugar Accumulation
Source: PLoS One. 2014 Nov 24;9(11):e113623. doi: 10.1371/journal.pone.0113623 (PMC4242728; doi:10.1371/journal.pone.0113623)
Supplement: Table S3 — Identity matrix for the amino acid sequences of six CitSus genes and three CuSuSy genes. (DOC) [file pone.0113623.s003.doc]

Table S3 Identity matrix for the amino acid sequences of six CitSus genes and three CuSuSy genes

|  | *CitSus2* | *CitSus3* | *CitSus4* | *CitSus5* | *CitSus6* | *CuSuSy1* | *CuSuSy2* | *CuSuSyA* |
| --- | --- | --- | --- | --- | --- | --- | --- | --- |
| *CitSus1* | 78.7 | 70.6 | 56.0 | 57.5 | 69.6 | 98.9 | 98.6 | 70.9 |
| *CitSus2* |  | 65.1 | 54.4 | 55.9 | 64.5 | 79.2 | 78.8 | 65.1 |
| *CitSus3* |  |  | 57.8 | 58.6 | 80.7 | 71.2 | 70.8 | 99.9 |
| *CitSus4* |  |  |  | 75.7 | 56.6 | 56.3 | 55.9 | 58.0 |
| *CitSus5* |  |  |  |  | 55.6 | 57.5 | 57.1 | 58.7 |
| *CitSus6* |  |  |  |  |  | 70.2 | 69.8 | 80.7 |
| *CuSuSy1* |  |  |  |  |  |  | 99.3 | 70.8 |
| *CuSuSy2* |  |  |  |  |  |  |  | 71.2 |
