# Supplementary material for: Genome-Wide Identification and Expression Profile Analysis of Citrus Sucrose Synthase Genes: Investigation of Possible Roles in the Regulation of Sugar Accumulation
Source: PLoS One. 2014 Nov 24;9(11):e113623. doi: 10.1371/journal.pone.0113623 (PMC4242728; doi:10.1371/journal.pone.0113623)
Supplement: Table S4 — Results of NCBI batch CD-search of six citrus sucrose synthases. (DOC) [file pone.0113623.s004.doc]

Table S4 Results of NCBI batch CD-search of six citrus sucrose synthases

| Query ID | Hit type | PSSM-ID | From | To | E-Value | Bitscore | Accession | Short name |
| --- | --- | --- | --- | --- | --- | --- | --- | --- |
| CitSus1 | specific | 99973 | 278 | 762 | 4.97E-126 | 386.126 | cd03800 | GT1_Sucrose_synthase |
|  | superfamily | 263942 | 278 | 762 | 4.97E-126 | 386.126 | cl10013 | Glycosyltransferase_GTB_type superfamily |
|  | multi-dom | 215073 | 1 | 805 | 0 | 1747.55 | PLN00142 | PLN00142 |
| CitSus2 | specific | 99973 | 252 | 735 | 6.65E-127 | 387.667 | cd03800 | GT1_Sucrose_synthase |
|  | superfamily | 263942 | 252 | 735 | 6.65E-127 | 387.667 | cl10013 | Glycosyltransferase_GTB_type superfamily |
|  | multi-dom | 215073 | 19 | 780 | 0 | 1531.45 | PLN00142 | PLN00142 |
| CitSus3 | specific | 99973 | 280 | 765 | 1.01E-135 | 411.164 | cd03800 | GT1_Sucrose_synthase |
|  | superfamily | 263942 | 280 | 765 | 1.01E-135 | 411.164 | cl10013 | Glycosyltransferase_GTB_type superfamily |
|  | multi-dom | 215073 | 1 | 811 | 0 | 1805.72 | PLN00142 | PLN00142 |
| CitSus4 | specific | 99973 | 282 | 766 | 1.49E-116 | 363.014 | cd03800 | GT1_Sucrose_synthase |
|  | superfamily | 263942 | 282 | 766 | 1.49E-116 | 363.014 | cl10013 | Glycosyltransferase_GTB_type superfamily |
|  | multi-dom | 215073 | 1 | 814 | 0 | 1683.99 | PLN00142 | PLN00142 |
| CitSus5 | specific | 99973 | 282 | 766 | 8.48E-118 | 365.325 | cd03800 | GT1_Sucrose_synthase |
|  | superfamily | 263942 | 282 | 766 | 8.48E-118 | 365.325 | cl10013 | Glycosyltransferase_GTB_type superfamily |
|  | multi-dom | 215073 | 1 | 815 | 0 | 1679.76 | PLN00142 | PLN00142 |
| CitSus6 | specific | 99973 | 277 | 762 | 1.05E-138 | 418.868 | cd03800 | GT1_Sucrose_synthase |
|  | superfamily | 263942 | 277 | 762 | 1.05E-138 | 418.868 | cl10013 | Glycosyltransferase_GTB_type superfamily |
|  | multi-dom | 215073 | 5 | 808 | 0 | 1745.24 | PLN00142 | PLN00142 |
